# [Mn(bpb)(DMAP)(NO)], an {Mn–NO}^6^ nitrosyl with *Z*′ = 8

**DOI:** 10.1107/S1600536811038669

**Published:** 2011-09-30

**Authors:** Marilyn M. Olmstead, A. Alegra Eroy-Reveles, Pradip K. Mascharak

**Affiliations:** a Department of Chemistry, One Shields Ave., University of California, Davis, CA 95616, USA; b Department of Chemistry, University of California, Santa Cruz, CA 95064, USA

## Abstract

The structure of the title compound octa­kis­{[4-(dimethyl­amino)­pyridine](nitros­yl)[*N*,*N*′-(*o*-phenyl­ene)bis­(pyridine-2-carboxamidato)]manganese(II)} ethanol hepta­solvate 3.5-hydrate, [Mn(C_18_H_12_N_4_O_2_)(C_7_H_10_N_2_)(NO)]_8_·7C_2_H_5_OH·3.5H_2_O, or 8[Mn(bpb)(DMAP)(NO)]·7EtOH·3.5H_2_O, is an unusual example of a structure with *Z*′ = 8. The tetra­dentate bpb ligand, together with the nitrosyl and dimethyl­amino­pyridine ligands, gives rise to a distorted octa­hedral coordination environment for the Mn(II) ion. The average Mn—N_(N=O)_ bond length is 1.631 (13) Å. The eight mol­ecules in the asymmetric unit differ mainly in the rotation of the DMAP pyridine plane with respect to a reference plane of the Mn and three N atoms, one of which is the N atom of the NO group. The dihedral angles between the normals to these planes range from a minimum of 28.0 (2)° to a maximum of 64.2 (2)°. There are also some differences in O—H⋯O hydrogen bonding inter­actions. For example, of the sixteen C=O acceptors, there are seven different inter­actions with EtOH donors and two inter­actions with H_2_O donors. The crystal studied was found to be a two-component twin, with a 179.9° rotation about the real axis [−0.535, 0.004, 1.000]. Due to the presence of a superlattice and, consequently, the large number of weak reflections, the refinement utilized rigid solvate groups and isotropic displacement parameters for all except the Mn atoms. H atoms were not located for hydrate molecules.

## Related literature

For related structures, see: Eroy-Reveles *et al.* (2008 ▶); Feng & Liao (2008 ▶); Ghosh *et al.* (2004 ▶); Hoffman-Luca *et al.* (2009 ▶); Liang *et al.* (2007 ▶). For {M—NO}^*x*^ formalism, see: Enemark & Feltham (1974 ▶). 
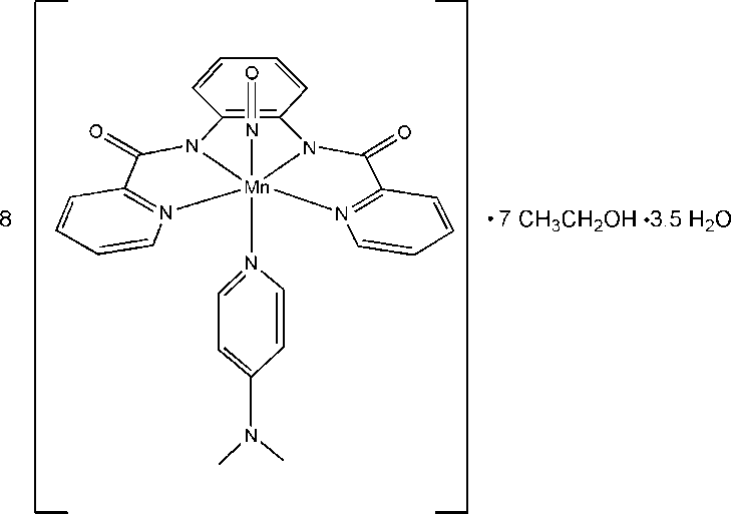


## Experimental

### 

#### Crystal data

[Mn(C_18_H_12_N_4_O_2_)(C_7_H_10_N_2_)(NO)]_8_·7C_2_H_6_O·3.5H_2_O
*M* *_r_* = 571.63
Monoclinic, 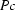

*a* = 24.943 (3) Å
*b* = 14.4343 (18) Å
*c* = 29.688 (4) Å
β = 101.190 (3)°
*V* = 10486 (2) Å^3^
*Z* = 16
Mo *K*α radiation
μ = 0.55 mm^−1^
*T* = 90 K
0.28 × 0.04 × 0.03 mm

#### Data collection

Bruker SMART APEXII diffractometer
Absorption correction: multi-scan (*TWINABS*; Sheldrick, 2005 ▶) *T*_min_ = 0.861, *T*_max_ = 0.984
158363 measured reflections
94844 independent reflections
26534 reflections with *I* > 2σ(*I*)
*R*_int_ = 0.164

#### Refinement

*R*[*F* ^2^ > 2σ(*F* ^2^)] = 0.068
*wR*(*F* ^2^) = 0.151
*S* = 0.64
19177 reflections
1275 parameters
3166 restraints
H-atom parameters constrained
Δρ_max_ = 1.04 e Å^−3^
Δρ_min_ = −0.55 e Å^−3^

### 

Data collection: *APEX2* (Bruker, 2005 ▶); cell refinement: *SAINT* (Bruker, 2005 ▶); data reduction: *SAINT*; program(s) used to solve structure: *SHELXS97* (Sheldrick, 2008 ▶); program(s) used to refine structure: *SHELXL97* (Sheldrick, 2008 ▶); molecular graphics: *XP* in *SHELXTL* (Sheldrick, 2008 ▶); software used to prepare material for publication: *SHELXL97* and *publCIF* (Westrip, 2010 ▶).

## Supplementary Material

Crystal structure: contains datablock(s) I, global. DOI: 10.1107/S1600536811038669/wm2529sup1.cif

Structure factors: contains datablock(s) I. DOI: 10.1107/S1600536811038669/wm2529Isup2.hkl

Additional supplementary materials:  crystallographic information; 3D view; checkCIF report

## Figures and Tables

**Table 1:** Hydrogen-bond geometry (Å, °)

*D*—H⋯*A*	*D*—H	H⋯*A*	*D*⋯*A*	*D*—H⋯*A*
O25—H20*D*⋯O22^i^	0.96	1.88	2.751 (15)	149
O26—H20*J*⋯O8^ii^	0.85	1.84	2.686 (17)	178
O27—H20*P*⋯O16^iii^	0.84	1.93	2.739 (18)	161
O28—H20*V*⋯O23	0.85	1.89	2.747 (19)	179
O29—H21*C*⋯O20	0.84	1.93	2.709 (17)	154
O30—H21*J*⋯O7^iv^	0.85	1.90	2.75 (2)	178
O31—H21*P*⋯O13	0.85	2.04	2.887 (17)	179

Symmetry codes: (i) 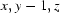; (ii) 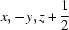; (iii) 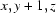; (iv) 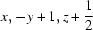.

**Fig. 1.**
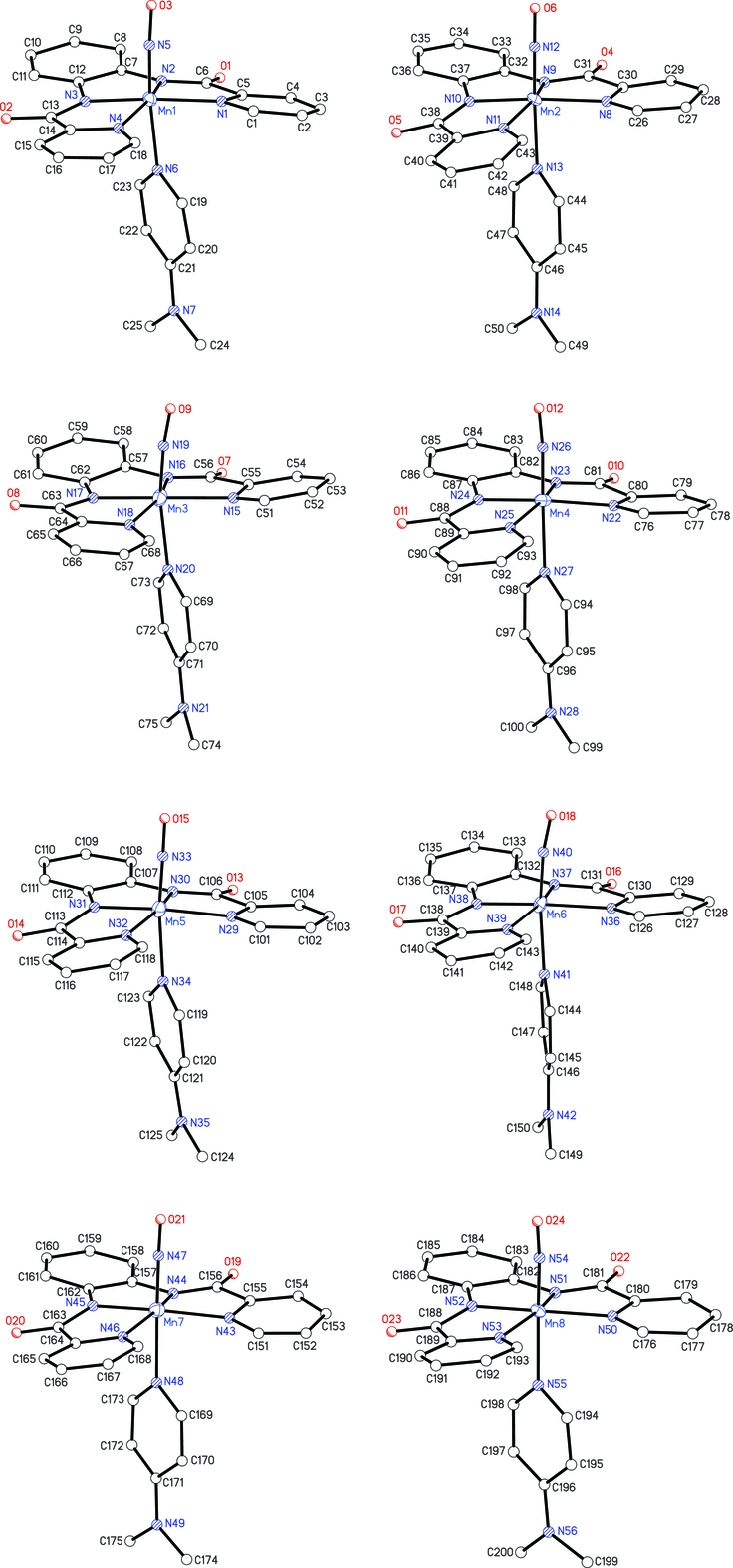
A view of the eight complexes in the asymmetric unit. The Mn atoms are shown with displacement parameters at the 35% probability level. Other atoms are assigned an arbitrary size. Ethanol and water molecules are not shown.

## supplementary crystallographic information

### Comment

The structure determination of the title compound, 8[Mn(C_18_H_12_N_4_O_2_)(C_7_H_10_N_2_)(NO)]^.^7C_2_H_5_OH^.^3.5H_2_O, was found to involve a rotational twin. The structure is unusual in that there are eight molecules of the complex, [Mn(bpb)(DMAP)(NO)], (bpb = *N*,*N*'-*o*-phenylenebis(pyridine-2-carboxamidate; DMAP = dimethylaminopyridine) in the asymmetric unit. The structure also contains seven ethanol and 3.5 water solvent molecules in the asymmetric unit. The bpb ligand occupies the four equatorial coordination sites of the manganese ion. The axial sites are occupied by a nitrosyl group and DMAP, completing a distorted octahedral coordination of the metal ion. The two Mn—N bonds from pyridyl groups are slightly longer at 2.064[13] Å than the two from amido groups (1.950[10] Å), (average with average deviation from the mean in square brackets). This trend has been seen in a number of similar complexes (Ghosh *et al.*, 2004; Liang *et al.*, 2007; Eroy-Reveles *et al.*, 2008; Feng & Liao, 2008; Hoffman-Luca, *et al.*, 2009). The average Mn—N_(DMAP)_ distance is 2.124[10] Å.

The geometry of the nitrosyl group does not vary a great deal from one complex to the next. It is nearly linear; the average Mn—N and N═O distances are 1.631[13] Å and 1.203[12] Å, respectively. The average Mn—N═O angle is 174.3[1.9] °. A notable difference concerns the rotation of the DMAP group, and this can be viewed in Fig. 1. In molecule 1, the angle between the normal to the plane consisting of Mn1/N2/N4/N5 and the normal to the pyridine plane N6/C19/C20/C21/C22/C23 is 51.6 (2)°. For each of the eight molecules, the same angle is calculated. For the other seven molecules these angles are: 2, 51.8 (2)°; 3, 39.4 (2)°; 4, 50.8 (2)°; 5, 44.4 (2)°; 6, 28.0 (2)°; 7, 58.0 (3)°; 8, 64.2 (3)°.

Hydrogen bonding interactions between the ethanol molecules and C═O groups of the bpb ligands are listed in Table 1. Additional hydrogen bonding that involves hydrate molecules is clearly present but not listed due to the difficulty in locating H atoms attached to the water molecules.

The oxidation state of Mn in the title compound, [Mn(bpb)(DMAP)(NO)], is most likely to be +II, *i.e.*, we can call the complex {Mn—NO}^6^ (Enemark & Feltham, 1974). This nitrosyl has its N═O stretching frequency at 1730 cm^-1^. A related complex of the deprotonated pentadentate ligand *N*,*N*-bis(2-pyridylmethyl)amine-*N*-ethyl-2-pyridine-2-carboxamide (PaPy_3_H), was previously reported [Mn(PaPy_3_)(NO)](ClO_4_) (Ghosh *et al.*, 2004). This is formally a Mn^II^—NO or Mn^I^—NO^+^ species, and the N═O stretching frequency appears at 1745 cm^-1^, while for the corresponding oxidized species [Mn(PaPy_3_)(NO)](ClO_4_)_2_, the N═O stretching frequency is at 1875 cm^-1^. Also, in the present complex the Mn—N_(O)_ distance is 1.631[13] Å. In [Mn(PaPy_3_)(NO)](ClO_4_), this distance is 1.6601 (14) Å. It is also important to note that the average Mn—N_(py)_ distance of the bpb ligand in the title complex is 2.064[13] Å while the same distance in [Mn(PaPy_3_)(NO)](ClO_4_) is 1.9953 (15) Å. It is evident that the present complex more closely resembles the Mn(II) species.

### Experimental

A portion of 100 mg (0.31 mmol) of the ligand bpbH_2_ was dissolved in 25 ml of degassed EtOH in a 100 ml Schlenk flask and to it was added a batch of 17 mg (0.65 mmol) of NaH. The pale yellow solution was stirred under nitrogen. Next, a batch of 86 mg (0.70 mmol) of DMAP (dimethylaminopyridine) was added to the solution and stirred. Once the solid was dissolved, the mixture was thoroughly degassed by two free-pump-thaw cycles. A batch of 114 mg (0.31 mmol) of [Mn(H_2_O)_6_](ClO_4_)_2_ was then added to the frozen mixture and it was allowed to thaw under dinitrogen. The color of the solution soon turned deep yellow and yellow solid separated out after 30 min. The headspace of the flask was then filled with NO(*g*) and the yellow-brown suspension was stirred under NO atmosphere for 2 h when the desired complex separated out as dark brown microcrystals. Anal. Calcd for [Mn(bpb)(DMAP)(NO)].EtOH.H_2_O, C_27_H_30_N_7_O_5_Mn: %C 55.20, %H 5.15, %N 16.69. Found: %C 55.32, %H 5.08, %N 16.51. Selected FTIR data (KBr matrix): νNO = 1730 cm^-1^. Crystals of [Mn(bpb)(DMAP)(NO)] suitable for X-ray studies, were grown from EtOH *via* slow evaporation.

### Refinement

The crystal was found to be a two-component twin, with a 179.9° rotation about the real axis [-0.535, 0.004, 1.000]. Integration was carried out with two components, and the absorption correction was applied with *TWINABS* (Sheldrick, 2005). For the HKLF 5 file, both components and composites were retained. The refined twin parameters were 0.32 (3) for the components and 0.12 (2) for and 0.08 (3) for racemic scale factors. In the final cycles of refinement, nextra was set to 75667 in order to better estimate the su's. Due to the large number of weak reflections in the dataset, it was not possible to refine all the atoms with anisotropic displacement parameters. Only the eight Mn atoms were assigned anisotropic displacement parameters. The seven molecules of ethanol were modeled using idealized geometry and treated as rigid groups in the final cycles of refinement. Three of the water molecules appeared to be at full occupancy, and a fourth was assigned the *ad hoc* value of 0.5 occupancy. The water hydrogen atoms could not be located with certainty and were not included in the model. Hydrogen atoms of the manganese complexes were added by geometry and refined as riding atoms with distances of H—C(*sp*^2^) = 0.95 and *H*—*C*(methyl) = 0.98 Å and with *U*_iso_(H) set to 1.2 (1.5 for methyl) *U*_eq_(C).

### Crystal data

**Table:**

[Mn(C_18_H_12_N_4_O_2_)(C_7_H_10_N_2_)(NO)]_8_·7C_2_H_6_O·3.5H_2_O	*F*(000) = 4754
*M**_r_* = 571.63	*D*_x_ = 1.448 Mg m^−^^3^
Monoclinic, *P**c*	Mo *K*α radiation, λ = 0.71073 Å
Hall symbol: P -2yc	Cell parameters from 3191 reflections
*a* = 24.943 (3) Å	θ = 2.2–20.6°
*b* = 14.4343 (18) Å	µ = 0.55 mm^−^^1^
*c* = 29.688 (4) Å	*T* = 90 K
β = 101.190 (3)°	Plate, brown
*V* = 10486 (2) Å^3^	0.28 × 0.04 × 0.03 mm
*Z* = 16	

### Data collection

**Table:**

Bruker SMART APEXII diffractometer	94844 independent reflections
Radiation source: fine-focus sealed tube	26534 reflections with *I* > 2σ(*I*)
graphite	*R*_int_ = 0.164
Detector resolution: 8.3 pixels mm^-1^	θ_max_ = 25.4°, θ_min_ = 1.7°
ω scans	*h* = −30→30
Absorption correction: multi-scan (*TWINABS*; Sheldrick, 2005)	*k* = −17→17
*T*_min_ = 0.861, *T*_max_ = 0.984	*l* = −35→35
158363 measured reflections	

### Refinement

**Table:**

Refinement on *F*^2^	Primary atom site location: structure-invariant direct methods
Least-squares matrix: full	Secondary atom site location: difference Fourier map
*R*[*F*^2^ > 2σ(*F*^2^)] = 0.068	Hydrogen site location: inferred from neighbouring sites
*wR*(*F*^2^) = 0.151	H-atom parameters constrained
*S* = 0.64	*w* = 1/[σ^2^(*F*_o_^2^) + (0.0236*P*)^2^] where *P* = (*F*_o_^2^ + 2*F*_c_^2^)/3
19177 reflections	(Δ/σ)_max_ = 0.079
1275 parameters	Δρ_max_ = 1.04 e Å^−^^3^
3166 restraints	Δρ_min_ = −0.55 e Å^−^^3^

### Special details

**Table:**

Geometry. All e.s.d.'s (except the e.s.d. in the dihedral angle between two l.s. planes) are estimated using the full covariance matrix. The cell e.s.d.'s are taken into account individually in the estimation of e.s.d.'s in distances, angles and torsion angles; correlations between e.s.d.'s in cell parameters are only used when they are defined by crystal symmetry. An approximate (isotropic) treatment of cell e.s.d.'s is used for estimating e.s.d.'s involving l.s. planes.
Refinement. Refinement of *F*^2^ against ALL reflections. The weighted *R*-factor *wR* and goodness of fit *S* are based on *F*^2^, conventional *R*-factors *R* are based on *F*, with *F* set to zero for negative *F*^2^. The threshold expression of *F*^2^ > σ(*F*^2^) is used only for calculating *R*-factors(gt) *etc*. and is not relevant to the choice of reflections for refinement. *R*-factors based on *F*^2^ are statistically about twice as large as those based on *F*, and *R*- factors based on ALL data will be even larger.

### Fractional atomic coordinates and isotropic or equivalent isotropic displacement parameters (Å^2^)

**Table:**

	*x*	*y*	*z*	*U*_iso_*/*U*_eq_	Occ. (<1)
Mn1	1.16133 (13)	0.9554 (2)	0.75887 (11)	0.0182 (9)	
O1	1.2040 (5)	1.2061 (8)	0.7169 (4)	0.022 (4)*	
O2	1.2120 (6)	0.7182 (9)	0.7044 (4)	0.032 (4)*	
O3	1.0498 (5)	0.9498 (9)	0.7141 (4)	0.026 (4)*	
N1	1.1457 (6)	1.0674 (9)	0.7954 (5)	0.021 (4)*	
N2	1.1838 (6)	1.0506 (9)	0.7197 (5)	0.023 (5)*	
N3	1.1924 (6)	0.8743 (9)	0.7187 (5)	0.016 (4)*	
N4	1.1579 (6)	0.8318 (8)	0.7935 (4)	0.013 (4)*	
N5	1.0977 (6)	0.9503 (10)	0.7323 (5)	0.023 (5)*	
N6	1.2412 (5)	0.9692 (9)	0.7998 (4)	0.012 (4)*	
N7	1.3994 (6)	1.0009 (10)	0.8792 (5)	0.016 (4)*	
C1	1.1252 (7)	1.0775 (11)	0.8343 (5)	0.016 (5)*	
H1	1.1119	1.0237	0.8469	0.019*	
C2	1.1224 (8)	1.1608 (11)	0.8569 (6)	0.028 (6)*	
H2	1.1078	1.1633	0.8841	0.034*	
C3	1.1410 (8)	1.2391 (13)	0.8393 (6)	0.029 (6)*	
H3	1.1401	1.2963	0.8550	0.034*	
C4	1.1612 (8)	1.2370 (12)	0.7991 (6)	0.025 (6)*	
H4	1.1728	1.2915	0.7858	0.030*	
C5	1.1634 (8)	1.1488 (11)	0.7789 (6)	0.021 (5)*	
C6	1.1857 (9)	1.1397 (12)	0.7345 (6)	0.029 (6)*	
C7	1.1992 (9)	1.0170 (11)	0.6808 (6)	0.015 (5)*	
C8	1.2100 (9)	1.0718 (13)	0.6432 (6)	0.034 (7)*	
H8	1.2066	1.1374	0.6428	0.041*	
C9	1.2262 (9)	1.0224 (12)	0.6064 (7)	0.032 (7)*	
H9	1.2321	1.0568	0.5805	0.038*	
C10	1.2340 (9)	0.9237 (13)	0.6059 (7)	0.039 (7)*	
H10	1.2457	0.8936	0.5811	0.046*	
C11	1.2239 (8)	0.8757 (12)	0.6429 (6)	0.021 (6)*	
H11	1.2297	0.8106	0.6439	0.025*	
C12	1.2049 (8)	0.9191 (11)	0.6802 (6)	0.017 (5)*	
C13	1.1959 (8)	0.7826 (11)	0.7286 (6)	0.026 (6)*	
C14	1.1732 (8)	0.7582 (11)	0.7694 (6)	0.016 (5)*	
C15	1.1710 (8)	0.6660 (12)	0.7833 (6)	0.028 (6)*	
H15	1.1827	0.6174	0.7659	0.034*	
C16	1.1522 (8)	0.6473 (12)	0.8213 (6)	0.028 (6)*	
H16	1.1483	0.5850	0.8305	0.034*	
C17	1.1381 (8)	0.7217 (12)	0.8478 (6)	0.027 (5)*	
H17	1.1267	0.7108	0.8760	0.032*	
C18	1.1416 (8)	0.8092 (12)	0.8316 (6)	0.022 (5)*	
H18	1.1312	0.8583	0.8494	0.026*	
C19	1.2511 (8)	0.9712 (12)	0.8461 (6)	0.023 (6)*	
H19	1.2209	0.9620	0.8608	0.028*	
C20	1.3005 (7)	0.9853 (13)	0.8730 (6)	0.022 (6)*	
H20	1.3036	0.9892	0.9053	0.026*	
C21	1.3477 (7)	0.9944 (13)	0.8537 (5)	0.015 (5)*	
C22	1.3379 (7)	0.9947 (13)	0.8063 (6)	0.023 (6)*	
H22	1.3674	1.0043	0.7908	0.028*	
C23	1.2857 (7)	0.9811 (13)	0.7812 (6)	0.020 (6)*	
H23	1.2810	0.9801	0.7487	0.024*	
C24	1.4076 (8)	1.0026 (14)	0.9286 (5)	0.023 (6)*	
H24A	1.3721	0.9994	0.9381	0.035*	
H24B	1.4262	1.0601	0.9401	0.035*	
H24C	1.4300	0.9494	0.9412	0.035*	
C25	1.4465 (7)	1.0031 (13)	0.8581 (6)	0.021 (6)*	
H25A	1.4349	1.0001	0.8246	0.031*	
H25B	1.4701	0.9501	0.8687	0.031*	
H25C	1.4668	1.0607	0.8665	0.031*	
Mn2	1.05533 (13)	1.4634 (2)	0.70853 (11)	0.0191 (9)	
O4	1.0729 (5)	1.2372 (8)	0.6295 (4)	0.031 (4)*	
O5	1.0601 (5)	1.7205 (9)	0.6538 (4)	0.041 (4)*	
O6	1.1569 (5)	1.4482 (9)	0.7702 (4)	0.031 (4)*	
N8	1.0300 (6)	1.3369 (8)	0.7292 (4)	0.007 (4)*	
N9	1.0714 (6)	1.3892 (9)	0.6584 (4)	0.020 (4)*	
N10	1.0732 (6)	1.5647 (9)	0.6708 (5)	0.025 (5)*	
N11	1.0316 (6)	1.5753 (9)	0.7427 (5)	0.019 (4)*	
N12	1.1137 (6)	1.4543 (10)	0.7433 (5)	0.018 (4)*	
N13	0.9752 (5)	1.4773 (10)	0.6706 (5)	0.022 (5)*	
N14	0.8138 (6)	1.5220 (10)	0.5973 (5)	0.020 (4)*	
C26	1.0097 (7)	1.3107 (11)	0.7656 (5)	0.016 (5)*	
H26	1.0022	1.3586	0.7854	0.019*	
C27	0.9984 (8)	1.2207 (11)	0.7776 (6)	0.021 (5)*	
H27	0.9824	1.2074	0.8034	0.025*	
C28	1.0128 (8)	1.1485 (12)	0.7481 (6)	0.025 (5)*	
H28	1.0083	1.0850	0.7549	0.030*	
C29	1.0332 (8)	1.1745 (12)	0.7096 (6)	0.036 (6)*	
H29	1.0421	1.1284	0.6894	0.044*	
C30	1.0407 (7)	1.2687 (11)	0.7003 (5)	0.015 (5)*	
C31	1.0644 (8)	1.2953 (11)	0.6581 (6)	0.023 (5)*	
C32	1.0962 (7)	1.4355 (11)	0.6273 (5)	0.015 (5)*	
C33	1.1166 (7)	1.3933 (12)	0.5906 (6)	0.026 (5)*	
H33	1.1135	1.3289	0.5839	0.031*	
C34	1.1417 (8)	1.4564 (12)	0.5653 (6)	0.030 (6)*	
H34	1.1575	1.4317	0.5412	0.036*	
C35	1.1458 (7)	1.5489 (12)	0.5720 (6)	0.027 (5)*	
H35	1.1634	1.5857	0.5526	0.032*	
C36	1.1239 (8)	1.5920 (13)	0.6076 (6)	0.046 (7)*	
H36A	1.1262	1.6571	0.6125	0.055*	
C37	1.0981 (7)	1.5331 (11)	0.6358 (6)	0.021 (5)*	
C38	1.0575 (7)	1.6486 (11)	0.6777 (5)	0.016 (5)*	
C39	1.0352 (8)	1.6566 (11)	0.7193 (6)	0.018 (5)*	
C40	1.0173 (8)	1.7386 (12)	0.7348 (6)	0.023 (5)*	
H40	1.0186	1.7941	0.7178	0.028*	
C41	0.9978 (9)	1.7405 (14)	0.7745 (7)	0.046 (7)*	
H41	0.9874	1.7981	0.7857	0.055*	
C42	0.9925 (7)	1.6597 (11)	0.7993 (6)	0.022 (5)*	
H4A	0.9772	1.6595	0.8262	0.026*	
C43	1.0115 (7)	1.5793 (11)	0.7815 (5)	0.020 (5)*	
H43	1.0101	1.5233	0.7980	0.023*	
C44	0.9316 (7)	1.4741 (12)	0.6905 (6)	0.016 (5)*	
H44	0.9379	1.4620	0.7226	0.019*	
C45	0.8782 (7)	1.4870 (11)	0.6680 (5)	0.012 (5)*	
H45	0.8496	1.4807	0.6848	0.014*	
C46	0.8652 (6)	1.5087 (12)	0.6222 (5)	0.016 (5)*	
C47	0.9117 (6)	1.5135 (11)	0.6007 (6)	0.014 (5)*	
H47	0.9067	1.5270	0.5688	0.016*	
C48	0.9629 (7)	1.4991 (12)	0.6251 (6)	0.025 (5)*	
H48	0.9924	1.5047	0.6094	0.030*	
C49	0.7681 (7)	1.5197 (13)	0.6224 (6)	0.027 (6)*	
H49A	0.7824	1.5068	0.6549	0.041*	
H49B	0.7423	1.4710	0.6095	0.041*	
H49C	0.7495	1.5798	0.6193	0.041*	
C50	0.8045 (7)	1.5414 (12)	0.5487 (5)	0.026 (5)*	
H50A	0.8396	1.5422	0.5384	0.038*	
H50B	0.7868	1.6020	0.5427	0.038*	
H50C	0.7810	1.4934	0.5319	0.038*	
Mn3	0.29953 (13)	0.2148 (2)	0.51169 (10)	0.0238 (9)	
O7	0.1781 (6)	0.3926 (9)	0.4549 (4)	0.034 (4)*	
O8	0.2765 (6)	−0.0687 (9)	0.4950 (5)	0.043 (4)*	
O9	0.2911 (6)	0.2230 (10)	0.6054 (4)	0.039 (4)*	
N15	0.3158 (6)	0.3573 (8)	0.5188 (5)	0.019 (4)*	
N16	0.2273 (5)	0.2614 (10)	0.4854 (5)	0.024 (5)*	
N17	0.2662 (6)	0.0932 (9)	0.4924 (5)	0.028 (5)*	
N18	0.3674 (5)	0.1272 (9)	0.5283 (5)	0.017 (4)*	
N19	0.2922 (7)	0.2155 (11)	0.5649 (5)	0.033 (5)*	
N20	0.3240 (6)	0.2342 (10)	0.4470 (4)	0.019 (4)*	
N21	0.3700 (6)	0.2714 (11)	0.3209 (5)	0.022 (4)*	
C51	0.3593 (8)	0.4005 (12)	0.5436 (6)	0.027 (6)*	
H51	0.3910	0.3664	0.5568	0.032*	
C52	0.3574 (7)	0.4972 (11)	0.5497 (6)	0.010 (5)*	
H52	0.3889	0.5274	0.5664	0.012*	
C53	0.3106 (8)	0.5517 (13)	0.5323 (7)	0.034 (6)*	
H53	0.3096	0.6165	0.5378	0.040*	
C54	0.2668 (8)	0.5055 (11)	0.5068 (6)	0.017 (5)*	
H54	0.2352	0.5389	0.4926	0.021*	
C55	0.2688 (7)	0.4072 (11)	0.5015 (7)	0.026 (6)*	
C56	0.2189 (8)	0.3521 (12)	0.4784 (7)	0.031 (6)*	
C57	0.1859 (8)	0.1916 (13)	0.4719 (7)	0.042 (7)*	
C58	0.1292 (9)	0.2093 (16)	0.4599 (8)	0.070 (9)*	
H58	0.1142	0.2696	0.4610	0.084*	
C59	0.0962 (9)	0.1297 (15)	0.4460 (7)	0.066 (8)*	
H59	0.0580	0.1371	0.4359	0.079*	
C60	0.1188 (9)	0.0408 (15)	0.4469 (7)	0.059 (8)*	
H60	0.0945	−0.0093	0.4375	0.071*	
C61	0.1743 (8)	0.0200 (13)	0.4605 (7)	0.037 (6)*	
H61	0.1879	−0.0417	0.4620	0.044*	
C62	0.2094 (7)	0.0997 (13)	0.4722 (7)	0.041 (7)*	
C63	0.2935 (7)	0.0159 (12)	0.5007 (7)	0.027 (6)*	
C64	0.3509 (7)	0.0354 (11)	0.5234 (6)	0.020 (5)*	
C65	0.3868 (7)	−0.0363 (12)	0.5348 (6)	0.025 (5)*	
H65	0.3752	−0.0990	0.5310	0.030*	
C66	0.4411 (8)	−0.0135 (13)	0.5522 (7)	0.042 (6)*	
H66	0.4667	−0.0620	0.5609	0.050*	
C67	0.4592 (7)	0.0787 (11)	0.5572 (6)	0.020 (5)*	
H67	0.4965	0.0939	0.5684	0.024*	
C68	0.4201 (7)	0.1455 (12)	0.5448 (6)	0.023 (6)*	
H68	0.4311	0.2085	0.5482	0.028*	
C69	0.3759 (7)	0.2533 (12)	0.4450 (6)	0.017 (5)*	
H69	0.4021	0.2595	0.4727	0.020*	
C70	0.3920 (8)	0.2641 (13)	0.4042 (5)	0.028 (6)*	
H70	0.4295	0.2744	0.4041	0.034*	
C71	0.3555 (7)	0.2605 (12)	0.3627 (5)	0.011 (5)*	
C72	0.2991 (7)	0.2479 (12)	0.3646 (6)	0.016 (5)*	
H72	0.2718	0.2482	0.3374	0.019*	
C73	0.2857 (7)	0.2355 (12)	0.4069 (5)	0.018 (5)*	
H73	0.2483	0.2275	0.4086	0.021*	
C74	0.4263 (7)	0.2735 (14)	0.3170 (6)	0.029 (6)*	
H74A	0.4491	0.2843	0.3473	0.043*	
H74B	0.4320	0.3235	0.2961	0.043*	
H74C	0.4362	0.2141	0.3049	0.043*	
C75	0.3299 (7)	0.2577 (13)	0.2786 (5)	0.019 (5)*	
H75A	0.2930	0.2581	0.2854	0.029*	
H75B	0.3365	0.1980	0.2649	0.029*	
H75C	0.3332	0.3076	0.2569	0.029*	
Mn4	0.54984 (13)	0.5246 (2)	0.47811 (10)	0.0200 (9)	
O10	0.4962 (6)	0.7669 (9)	0.5250 (4)	0.032 (4)*	
O11	0.5037 (6)	0.2800 (9)	0.5276 (4)	0.040 (4)*	
O12	0.6608 (6)	0.5207 (9)	0.5241 (5)	0.035 (4)*	
N22	0.5555 (6)	0.6439 (8)	0.4426 (4)	0.012 (4)*	
N23	0.5231 (6)	0.6133 (9)	0.5182 (4)	0.016 (4)*	
N24	0.5253 (6)	0.4355 (9)	0.5190 (4)	0.013 (4)*	
N25	0.5604 (6)	0.4040 (8)	0.4427 (4)	0.014 (4)*	
N26	0.6143 (6)	0.5258 (10)	0.5035 (5)	0.021 (5)*	
N27	0.4701 (5)	0.5163 (10)	0.4370 (5)	0.022 (5)*	
N28	0.3132 (6)	0.5041 (11)	0.3555 (5)	0.016 (4)*	
C76	0.5772 (8)	0.6601 (12)	0.4052 (6)	0.021 (5)*	
H76	0.5898	0.6082	0.3906	0.025*	
C77	0.5825 (8)	0.7486 (11)	0.3860 (6)	0.027 (6)*	
H77	0.5994	0.7565	0.3602	0.032*	
C78	0.5619 (8)	0.8231 (12)	0.4067 (6)	0.023 (5)*	
H78	0.5625	0.8836	0.3941	0.027*	
C79	0.5406 (7)	0.8091 (11)	0.4455 (5)	0.013 (5)*	
H79	0.5285	0.8602	0.4610	0.015*	
C80	0.5368 (9)	0.7169 (12)	0.4625 (6)	0.031 (6)*	
C81	0.5174 (8)	0.7030 (11)	0.5065 (6)	0.020 (5)*	
C82	0.5090 (8)	0.5732 (11)	0.5572 (6)	0.018 (5)*	
C83	0.4965 (8)	0.6205 (12)	0.5949 (6)	0.026 (6)*	
H83	0.4976	0.6863	0.5956	0.031*	
C84	0.4825 (8)	0.5717 (12)	0.6319 (6)	0.022 (6)*	
H84	0.4722	0.6046	0.6566	0.026*	
C85	0.4836 (8)	0.4767 (11)	0.6324 (6)	0.017 (6)*	
H85	0.4756	0.4449	0.6584	0.021*	
C86	0.4964 (8)	0.4246 (12)	0.5955 (6)	0.026 (6)*	
H86	0.4953	0.3588	0.5956	0.031*	
C87	0.5110 (9)	0.4742 (11)	0.5582 (6)	0.020 (6)*	
C88	0.5220 (9)	0.3461 (12)	0.5071 (6)	0.027 (6)*	
C89	0.5420 (8)	0.3301 (11)	0.4641 (6)	0.022 (6)*	
C90	0.5435 (8)	0.2436 (12)	0.4474 (6)	0.029 (6)*	
H90	0.5316	0.1926	0.4632	0.035*	
C91	0.5626 (7)	0.2293 (12)	0.4068 (6)	0.025 (5)*	
H91	0.5640	0.1687	0.3947	0.030*	
C92	0.5798 (7)	0.3064 (11)	0.3840 (6)	0.016 (5)*	
H92	0.5921	0.2996	0.3559	0.019*	
C93	0.5780 (8)	0.3924 (13)	0.4045 (6)	0.037 (6)*	
H93	0.5902	0.4451	0.3901	0.044*	
C94	0.4622 (7)	0.5138 (12)	0.3905 (5)	0.016 (5)*	
H94	0.4932	0.5170	0.3764	0.019*	
C95	0.4115 (7)	0.5069 (13)	0.3629 (6)	0.014 (5)*	
H95	0.4088	0.5031	0.3306	0.016*	
C96	0.3633 (7)	0.5054 (14)	0.3811 (5)	0.015 (5)*	
C97	0.3748 (7)	0.5025 (13)	0.4289 (5)	0.014 (5)*	
H97	0.3452	0.4944	0.4444	0.016*	
C98	0.4260 (7)	0.5106 (13)	0.4546 (6)	0.020 (5)*	
H98	0.4300	0.5123	0.4871	0.023*	
C99	0.3037 (8)	0.5038 (14)	0.3063 (6)	0.024 (6)*	
H99A	0.3387	0.4989	0.2961	0.036*	
H99B	0.2854	0.5614	0.2946	0.036*	
H99C	0.2805	0.4508	0.2946	0.036*	
C100	0.2656 (8)	0.5064 (14)	0.3780 (7)	0.031 (6)*	
H10B	0.2320	0.5021	0.3547	0.047*	
H10C	0.2657	0.5646	0.3950	0.047*	
H10D	0.2675	0.4541	0.3993	0.047*	
Mn5	0.65198 (14)	1.0340 (2)	0.52464 (11)	0.0229 (10)	
O13	0.6322 (5)	0.8118 (8)	0.6036 (4)	0.028 (4)*	
O14	0.6501 (5)	1.2919 (9)	0.5800 (4)	0.037 (4)*	
O15	0.5565 (5)	1.0268 (9)	0.4557 (4)	0.030 (4)*	
N29	0.6767 (6)	0.9055 (9)	0.5062 (5)	0.025 (5)*	
N30	0.6276 (6)	0.9600 (8)	0.5711 (4)	0.016 (4)*	
N31	0.6325 (6)	1.1352 (9)	0.5619 (4)	0.016 (4)*	
N32	0.6817 (6)	1.1411 (9)	0.4917 (5)	0.023 (4)*	
N33	0.5960 (6)	1.0275 (10)	0.4864 (5)	0.019 (5)*	
N34	0.7322 (5)	1.0344 (10)	0.5646 (5)	0.016 (5)*	
N35	0.8882 (6)	1.0084 (10)	0.6471 (5)	0.022 (4)*	
C101	0.6992 (7)	0.8808 (11)	0.4722 (6)	0.020 (5)*	
H101	0.7085	0.9282	0.4528	0.024*	
C102	0.7109 (8)	0.7879 (12)	0.4619 (6)	0.024 (6)*	
H102	0.7273	0.7724	0.4366	0.029*	
C103	0.6966 (7)	0.7206 (11)	0.4912 (5)	0.013 (5)*	
H103	0.7035	0.6571	0.4860	0.016*	
C104	0.6725 (7)	0.7447 (10)	0.5280 (5)	0.012 (5)*	
H104	0.6618	0.6989	0.5474	0.015*	
C105	0.6647 (8)	0.8387 (11)	0.5352 (6)	0.024 (6)*	
C106	0.6382 (7)	0.8694 (10)	0.5750 (5)	0.012 (5)*	
C107	0.6040 (8)	1.0132 (11)	0.6033 (6)	0.022 (5)*	
C108	0.5763 (8)	0.9757 (13)	0.6362 (6)	0.043 (6)*	
H108	0.5720	0.9108	0.6395	0.052*	
C109	0.5546 (8)	1.0423 (11)	0.6645 (6)	0.021 (6)*	
H109	0.5378	1.0196	0.6884	0.025*	
C110	0.5571 (8)	1.1353 (12)	0.6587 (6)	0.034 (6)*	
H110	0.5416	1.1761	0.6779	0.041*	
C111	0.5825 (7)	1.1706 (13)	0.6245 (6)	0.034 (6)*	
H111	0.5831	1.2356	0.6195	0.041*	
C112	0.6072 (8)	1.1104 (12)	0.5974 (6)	0.032 (6)*	
C113	0.6526 (8)	1.2200 (12)	0.5554 (6)	0.025 (5)*	
C114	0.6788 (7)	1.2230 (11)	0.5143 (5)	0.019 (5)*	
C115	0.6947 (8)	1.3075 (12)	0.4998 (6)	0.026 (6)*	
H115	0.6898	1.3630	0.5157	0.032*	
C116	0.7185 (8)	1.3086 (13)	0.4605 (6)	0.034 (6)*	
H116	0.7323	1.3644	0.4502	0.041*	
C117	0.7212 (8)	1.2236 (12)	0.4368 (6)	0.037 (6)*	
H117	0.7350	1.2218	0.4091	0.044*	
C118	0.7035 (7)	1.1442 (12)	0.4548 (6)	0.025 (5)*	
H118	0.7073	1.0874	0.4395	0.030*	
C119	0.7773 (7)	1.0340 (12)	0.5471 (6)	0.023 (6)*	
H119	0.7732	1.0371	0.5147	0.027*	
C120	0.8296 (7)	1.0293 (12)	0.5726 (5)	0.018 (5)*	
H120	0.8601	1.0329	0.5579	0.021*	
C121	0.8377 (6)	1.0191 (12)	0.6209 (5)	0.015 (5)*	
C122	0.7905 (6)	1.0244 (12)	0.6396 (6)	0.020 (5)*	
H122	0.7932	1.0208	0.6720	0.023*	
C123	0.7408 (7)	1.0346 (12)	0.6117 (5)	0.026 (6)*	
H123	0.7099	1.0425	0.6256	0.032*	
C124	0.9356 (7)	1.0031 (12)	0.6254 (6)	0.013 (5)*	
H12D	0.9244	1.0153	0.5924	0.020*	
H12E	0.9518	0.9411	0.6300	0.020*	
H12F	0.9627	1.0494	0.6391	0.020*	
C125	0.8955 (8)	0.9973 (13)	0.6962 (5)	0.033 (6)*	
H12G	0.9346	0.9938	0.7095	0.049*	
H12H	0.8775	0.9402	0.7031	0.049*	
H12I	0.8794	1.0503	0.7093	0.049*	
Mn6	0.76186 (12)	0.2699 (2)	0.75693 (10)	0.0207 (8)	
O16	0.8851 (5)	0.0955 (8)	0.8075 (4)	0.027 (4)*	
O17	0.7896 (6)	0.5496 (9)	0.7533 (4)	0.030 (4)*	
O18	0.6994 (5)	0.2506 (8)	0.8257 (4)	0.032 (4)*	
N36	0.7484 (6)	0.1305 (8)	0.7459 (5)	0.025 (4)*	
N37	0.8296 (5)	0.2237 (9)	0.7942 (4)	0.019 (4)*	
N38	0.7968 (6)	0.3888 (8)	0.7701 (5)	0.021 (4)*	
N39	0.7034 (6)	0.3519 (9)	0.7172 (5)	0.023 (4)*	
N40	0.7269 (5)	0.2649 (9)	0.7978 (4)	0.014 (4)*	
N41	0.7989 (5)	0.2630 (9)	0.6982 (4)	0.013 (4)*	
N42	0.8757 (6)	0.2560 (10)	0.5832 (4)	0.025 (4)*	
C126	0.7035 (8)	0.0857 (12)	0.7240 (6)	0.032 (6)*	
H126	0.6734	0.1197	0.7074	0.038*	
C127	0.7013 (8)	−0.0121 (12)	0.7257 (6)	0.033 (6)*	
H127	0.6685	−0.0426	0.7120	0.039*	
C128	0.7462 (7)	−0.0654 (13)	0.7470 (6)	0.032 (6)*	
H128	0.7452	−0.1312	0.7464	0.039*	
C129	0.7927 (8)	−0.0173 (11)	0.7694 (6)	0.024 (6)*	
H129	0.8238	−0.0497	0.7853	0.029*	
C130	0.7922 (7)	0.0822 (11)	0.7677 (6)	0.015 (5)*	
C131	0.8420 (7)	0.1359 (11)	0.7925 (6)	0.029 (6)*	
C132	0.8648 (6)	0.2918 (10)	0.8158 (5)	0.011 (4)*	
C133	0.9102 (7)	0.2757 (12)	0.8488 (5)	0.024 (5)*	
H133	0.9193	0.2148	0.8599	0.028*	
C134	0.9434 (8)	0.3524 (12)	0.8661 (6)	0.033 (6)*	
H134	0.9767	0.3424	0.8873	0.040*	
C135	0.9272 (7)	0.4449 (12)	0.8520 (6)	0.027 (6)*	
H135	0.9487	0.4955	0.8657	0.032*	
C136	0.8806 (7)	0.4623 (12)	0.8186 (6)	0.030 (6)*	
H136	0.8706	0.5233	0.8082	0.036*	
C137	0.8487 (7)	0.3838 (11)	0.8010 (6)	0.025 (5)*	
C138	0.7736 (7)	0.4645 (11)	0.7495 (6)	0.021 (5)*	
C139	0.7184 (6)	0.4414 (11)	0.7202 (5)	0.015 (5)*	
C140	0.6848 (7)	0.5115 (13)	0.6984 (6)	0.034 (6)*	
H140	0.6949	0.5749	0.7023	0.040*	
C141	0.6364 (8)	0.4846 (14)	0.6710 (7)	0.052 (7)*	
H141	0.6146	0.5287	0.6518	0.062*	
C142	0.6187 (8)	0.3913 (13)	0.6712 (6)	0.042 (6)*	
H142	0.5829	0.3737	0.6565	0.050*	
C143	0.6546 (7)	0.3280 (12)	0.6932 (6)	0.031 (6)*	
H143	0.6445	0.2644	0.6914	0.037*	
C144	0.7709 (7)	0.2633 (12)	0.6543 (5)	0.019 (5)*	
H144	0.7322	0.2639	0.6498	0.023*	
C145	0.7936 (7)	0.2629 (12)	0.6151 (5)	0.024 (5)*	
H145	0.7708	0.2675	0.5856	0.029*	
C146	0.8502 (7)	0.2557 (15)	0.6195 (6)	0.017 (4)*	
C147	0.8791 (7)	0.2540 (11)	0.6642 (5)	0.021 (5)*	
H147	0.9178	0.2497	0.6694	0.025*	
C148	0.8537 (7)	0.2584 (13)	0.7013 (6)	0.027 (6)*	
H148	0.8761	0.2583	0.7311	0.033*	
C149	0.8437 (7)	0.2641 (14)	0.5360 (5)	0.032 (6)*	
H14J	0.8683	0.2612	0.5141	0.048*	
H14K	0.8241	0.3233	0.5327	0.048*	
H14L	0.8173	0.2130	0.5301	0.048*	
C150	0.9348 (6)	0.2651 (13)	0.5877 (6)	0.025 (5)*	
H15B	0.9443	0.2624	0.5572	0.038*	
H15C	0.9530	0.2145	0.6067	0.038*	
H15D	0.9466	0.3246	0.6022	0.038*	
Mn7	0.94975 (12)	0.18291 (19)	0.99417 (10)	0.0211 (8)	
O19	0.9299 (6)	−0.0997 (9)	1.0005 (4)	0.039 (4)*	
O20	0.8235 (5)	0.3430 (8)	0.9297 (4)	0.028 (4)*	
O21	1.0195 (5)	0.1780 (7)	0.9291 (4)	0.023 (3)*	
N43	1.0043 (5)	0.1079 (9)	1.0425 (4)	0.022 (4)*	
N44	0.9165 (6)	0.0568 (9)	0.9853 (5)	0.019 (4)*	
N45	0.8836 (5)	0.2209 (9)	0.9516 (4)	0.024 (4)*	
N46	0.9592 (6)	0.3255 (8)	1.0001 (5)	0.025 (4)*	
N47	0.9893 (6)	0.1773 (9)	0.9545 (4)	0.016 (4)*	
N48	0.9073 (5)	0.2051 (9)	1.0491 (4)	0.016 (4)*	
N49	0.8244 (6)	0.2470 (10)	1.1580 (4)	0.025 (4)*	
C151	1.0478 (7)	0.1347 (12)	1.0741 (6)	0.029 (6)*	
H151	1.0565	0.1987	1.0778	0.034*	
C152	1.0795 (7)	0.0714 (12)	1.1008 (6)	0.034 (6)*	
H152	1.1081	0.0920	1.1247	0.041*	
C153	1.0706 (8)	−0.0230 (13)	1.0938 (6)	0.043 (6)*	
H153	1.0944	−0.0661	1.1118	0.052*	
C154	1.0279 (7)	−0.0550 (12)	1.0613 (6)	0.026 (5)*	
H154	1.0215	−0.1191	1.0554	0.031*	
C155	0.9946 (7)	0.0137 (11)	1.0376 (5)	0.020 (5)*	
C156	0.9425 (8)	−0.0178 (12)	1.0046 (6)	0.032 (6)*	
C157	0.8689 (7)	0.0579 (10)	0.9503 (5)	0.018 (5)*	
C158	0.8403 (7)	−0.0243 (12)	0.9321 (6)	0.023 (5)*	
H158	0.8516	−0.0840	0.9437	0.027*	
C159	0.7942 (7)	−0.0121 (11)	0.8959 (5)	0.014 (5)*	
H159	0.7748	−0.0656	0.8831	0.017*	
C160	0.7761 (8)	0.0748 (12)	0.8784 (6)	0.034 (6)*	
H160	0.7444	0.0795	0.8548	0.040*	
C161	0.8043 (7)	0.1542 (12)	0.8955 (6)	0.032 (6)*	
H161	0.7936	0.2132	0.8827	0.038*	
C162	0.8497 (6)	0.1454 (10)	0.9324 (5)	0.008 (4)*	
C163	0.8683 (7)	0.3104 (11)	0.9507 (6)	0.017 (5)*	
C164	0.9127 (7)	0.3710 (11)	0.9767 (6)	0.025 (5)*	
C165	0.9107 (9)	0.4687 (12)	0.9737 (7)	0.040 (7)*	
H165	0.8793	0.4987	0.9567	0.048*	
C166	0.9542 (7)	0.5195 (12)	0.9956 (6)	0.030 (5)*	
H166	0.9530	0.5853	0.9943	0.036*	
C167	0.9991 (8)	0.4756 (12)	1.0192 (6)	0.026 (6)*	
H167	1.0288	0.5111	1.0352	0.031*	
C168	1.0022 (7)	0.3790 (11)	1.0201 (6)	0.027 (6)*	
H168	1.0351	0.3500	1.0351	0.033*	
C169	0.9338 (8)	0.2354 (12)	1.0916 (5)	0.031 (6)*	
H169	0.9720	0.2468	1.0961	0.037*	
C170	0.9082 (6)	0.2495 (11)	1.1269 (6)	0.024 (5)*	
H170	0.9289	0.2688	1.1556	0.029*	
C171	0.8518 (7)	0.2365 (12)	1.1223 (5)	0.013 (4)*	
C172	0.8250 (7)	0.2033 (11)	1.0802 (5)	0.023 (5)*	
H172	0.7870	0.1904	1.0750	0.027*	
C173	0.8536 (6)	0.1895 (11)	1.0464 (5)	0.015 (5)*	
H173	0.8338	0.1664	1.0181	0.018*	
C174	0.8531 (7)	0.2715 (12)	1.2043 (5)	0.024 (5)*	
H174	0.8921	0.2798	1.2043	0.036*	
H17F	0.8484	0.2219	1.2257	0.036*	
H17G	0.8381	0.3294	1.2139	0.036*	
C175	0.7658 (6)	0.2302 (12)	1.1519 (5)	0.020 (5)*	
H17H	0.7511	0.2155	1.1197	0.030*	
H17I	0.7477	0.2857	1.1607	0.030*	
H17J	0.7592	0.1781	1.1713	0.030*	
Mn8	0.40525 (12)	0.2861 (2)	0.71914 (10)	0.0155 (8)	
O22	0.4102 (5)	0.5681 (8)	0.7363 (4)	0.017 (3)*	
O23	0.5350 (5)	0.1283 (8)	0.7780 (4)	0.025 (4)*	
O24	0.4176 (6)	0.2747 (9)	0.6270 (4)	0.036 (4)*	
N50	0.3334 (5)	0.3592 (9)	0.7019 (5)	0.015 (4)*	
N51	0.4321 (5)	0.4098 (8)	0.7389 (5)	0.010 (4)*	
N52	0.4801 (5)	0.2514 (9)	0.7465 (4)	0.006 (4)*	
N53	0.3980 (6)	0.1439 (8)	0.7156 (5)	0.012 (4)*	
N54	0.4133 (6)	0.2876 (9)	0.6666 (4)	0.014 (4)*	
N55	0.3843 (5)	0.2639 (10)	0.7838 (4)	0.011 (4)*	
N56	0.3342 (6)	0.2341 (11)	0.9105 (5)	0.018 (4)*	
C176	0.2817 (7)	0.3347 (12)	0.6866 (6)	0.020 (5)*	
H176	0.2750	0.2701	0.6825	0.024*	
C177	0.2369 (7)	0.3906 (12)	0.6761 (6)	0.027 (6)*	
H177	0.2013	0.3661	0.6660	0.032*	
C178	0.2465 (7)	0.4890 (11)	0.6811 (6)	0.018 (5)*	
H178	0.2171	0.5316	0.6736	0.021*	
C179	0.2994 (6)	0.5202 (11)	0.6969 (5)	0.009 (4)*	
H179	0.3076	0.5845	0.7000	0.011*	
C180	0.3409 (6)	0.4519 (11)	0.7084 (6)	0.015 (5)*	
C181	0.3991 (7)	0.4843 (11)	0.7295 (6)	0.018 (5)*	
C182	0.4898 (6)	0.4131 (10)	0.7558 (6)	0.008 (5)*	
C183	0.5199 (7)	0.4952 (11)	0.7696 (6)	0.013 (5)*	
H183	0.5026	0.5541	0.7679	0.015*	
C184	0.5758 (7)	0.4853 (11)	0.7857 (6)	0.018 (5)*	
H18D	0.5971	0.5387	0.7955	0.021*	
C185	0.6012 (7)	0.3996 (11)	0.7879 (6)	0.019 (5)*	
H185	0.6398	0.3971	0.7977	0.023*	
C186	0.5736 (6)	0.3186 (11)	0.7767 (5)	0.014 (5)*	
H186	0.5917	0.2603	0.7797	0.017*	
C187	0.5170 (7)	0.3259 (11)	0.7603 (6)	0.020 (6)*	
C188	0.4909 (7)	0.1642 (11)	0.7569 (6)	0.020 (5)*	
C189	0.4454 (7)	0.1005 (11)	0.7357 (6)	0.012 (5)*	
C190	0.4493 (7)	0.0084 (11)	0.7343 (6)	0.015 (5)*	
H190	0.4820	−0.0207	0.7495	0.018*	
C191	0.4067 (7)	−0.0459 (12)	0.7113 (6)	0.016 (5)*	
H191	0.4099	−0.1114	0.7100	0.019*	
C192	0.3589 (8)	−0.0002 (12)	0.6901 (6)	0.019 (5)*	
H192	0.3287	−0.0341	0.6735	0.022*	
C193	0.3563 (7)	0.0951 (11)	0.6938 (6)	0.015 (5)*	
H193	0.3236	0.1262	0.6803	0.018*	
C194	0.3321 (7)	0.2399 (12)	0.7866 (5)	0.013 (5)*	
H194	0.3065	0.2288	0.7589	0.016*	
C195	0.3150 (7)	0.2311 (12)	0.8273 (5)	0.011 (5)*	
H195	0.2777	0.2171	0.8269	0.013*	
C196	0.3508 (7)	0.2421 (13)	0.8698 (5)	0.016 (5)*	
C197	0.4049 (7)	0.2640 (13)	0.8674 (6)	0.026 (6)*	
H197	0.4319	0.2709	0.8945	0.031*	
C198	0.4176 (7)	0.2751 (12)	0.8245 (5)	0.019 (5)*	
H198	0.4542	0.2927	0.8238	0.022*	
C199	0.2753 (7)	0.2260 (15)	0.9103 (7)	0.041 (7)*	
H19J	0.2554	0.2176	0.8787	0.061*	
H19K	0.2625	0.2825	0.9232	0.061*	
H19L	0.2688	0.1726	0.9289	0.061*	
C200	0.3712 (7)	0.2454 (14)	0.9543 (6)	0.031 (6)*	
H20A	0.4089	0.2497	0.9493	0.046*	
H20B	0.3680	0.1919	0.9739	0.046*	
H20C	0.3619	0.3021	0.9692	0.046*	
O25	0.4001 (4)	−0.2744 (7)	0.6831 (3)	0.025 (3)*	
C201	0.3580 (5)	−0.2891 (10)	0.6437 (4)	0.033 (6)*	
C202	0.3672 (7)	−0.2217 (11)	0.6068 (3)	0.024 (5)*	
H20D	0.3952	−0.3166	0.7072	0.029*	
H20E	0.3176	−0.2778	0.6523	0.029*	
H20F	0.3593	−0.3608	0.6314	0.029*	
H20G	0.3359	−0.2304	0.5757	0.029*	
H20H	0.3657	−0.1503	0.6187	0.029*	
H20I	0.4072	−0.2331	0.5978	0.029*	
O26	0.2449 (4)	0.2389 (8)	1.0157 (4)	0.027 (3)*	
C203	0.1915 (4)	0.2476 (11)	0.9879 (4)	0.036 (6)*	
C204	0.1508 (4)	0.2097 (12)	1.0150 (5)	0.036 (6)*	
H20J	0.2555	0.1854	1.0092	0.044*	
H20K	0.1890	0.2088	0.9557	0.044*	
H20L	0.1825	0.3206	0.9790	0.044*	
H20M	0.1091	0.2148	0.9951	0.044*	
H20N	0.1594	0.1369	1.0237	0.047*	
H20O	0.1529	0.2483	1.0470	0.044*	
O27	0.9582 (6)	0.9557 (9)	0.8031 (4)	0.042 (5)*	
C205	0.9927 (5)	0.9722 (12)	0.8468 (4)	0.042 (7)*	
C206	0.9597 (8)	0.9527 (13)	0.8836 (4)	0.047 (7)*	
H20P	0.9311	0.9914	0.7998	0.056*	
H20Q	1.0071	1.0443	0.8491	0.056*	
H20R	1.0288	0.9270	0.8514	0.056*	
H20S	0.9844	0.9644	0.9177	0.056*	
H20T	0.9239	0.9979	0.8793	0.056*	
H20U	0.9455	0.8809	0.8816	0.056*	
O28	0.5659 (6)	−0.0216 (9)	0.8341 (5)	0.056 (5)*	
C207	0.6229 (6)	−0.0043 (10)	0.8366 (7)	0.078 (9)*	
C208	0.6486 (7)	−0.0922 (14)	0.8228 (8)	0.099 (10)*	
H20V	0.5559	0.0251	0.8169	0.119*	
H20W	0.6423	0.0159	0.8717	0.119*	
H20X	0.6287	0.0530	0.8136	0.119*	
H20Y	0.6923	−0.0824	0.8242	0.119*	
H21A	0.6432	−0.1492	0.8457	0.119*	
H21B	0.6296	−0.1122	0.7878	0.119*	
O29	0.7486 (6)	0.4730 (8)	0.9401 (4)	0.033 (4)*	
C209	0.7173 (5)	0.4668 (11)	0.8945 (4)	0.040 (7)*	
C21F	0.7372 (8)	0.5019 (13)	0.8595 (4)	0.045 (7)*	
H21C	0.7785	0.4452	0.9415	0.054*	
H21D	0.7097	0.4004	0.8878	0.054*	
H21E	0.6817	0.4972	0.8944	0.054*	
H21G	0.7109	0.4919	0.8308	0.054*	
H21H	0.7717	0.4710	0.8577	0.054*	
H21I	0.7436	0.5684	0.8643	0.054*	
O30	0.1495 (6)	0.4560 (10)	0.8991 (5)	0.061 (6)*	
C211	0.0914 (6)	0.4647 (16)	0.8920 (5)	0.081 (10)*	
C212	0.0741 (8)	0.4444 (16)	0.9371 (7)	0.093 (10)*	
H21J	0.1589	0.5023	0.9165	0.111*	
H21K	0.0718	0.4159	0.8654	0.111*	
H21L	0.0788	0.5350	0.8802	0.111*	
H21M	0.0297	0.4502	0.9334	0.111*	
H21N	0.0862	0.3743	0.9489	0.111*	
H21O	0.0932	0.4931	0.9636	0.111*	
O31	0.6384 (6)	0.7631 (9)	0.6988 (5)	0.059 (5)*	
C213	0.5881 (7)	0.7158 (11)	0.6988 (7)	0.091 (9)*	
C214	0.5545 (8)	0.7745 (19)	0.7253 (9)	0.161 (15)*	
H21P	0.6371	0.7769	0.6707	0.193*	
H21Q	0.5959	0.6473	0.7148	0.193*	
H21R	0.5658	0.7052	0.6634	0.193*	
H21S	0.5154	0.7411	0.7263	0.193*	
H21T	0.5763	0.7848	0.7606	0.193*	
H21U	0.5463	0.8426	0.7093	0.193*	
O1W	0.7376 (8)	0.7106 (13)	0.7660 (6)	0.106 (7)*	
O2W	0.7816 (8)	1.2427 (14)	0.3413 (7)	0.144 (8)*	
O3W	0.9659 (9)	0.7383 (14)	0.9704 (7)	0.137 (9)*	
O4W	0.8654 (14)	1.290 (2)	0.3861 (11)	0.092 (12)*	0.50

### Atomic displacement parameters (Å^2^)

**Table:**

	*U*^11^	*U*^22^	*U*^33^	*U*^12^	*U*^13^	*U*^23^
Mn1	0.016 (2)	0.0150 (19)	0.023 (2)	−0.0001 (16)	0.0020 (17)	−0.0018 (15)
Mn2	0.016 (2)	0.019 (2)	0.019 (2)	0.0029 (16)	−0.0028 (18)	0.0059 (16)
Mn3	0.032 (3)	0.021 (2)	0.018 (2)	−0.0079 (18)	0.0026 (18)	0.0002 (16)
Mn4	0.016 (2)	0.025 (2)	0.018 (2)	−0.0020 (16)	0.0000 (17)	−0.0014 (16)
Mn5	0.022 (3)	0.023 (2)	0.024 (2)	0.0039 (18)	0.0067 (19)	−0.0044 (17)
Mn6	0.022 (2)	0.0148 (18)	0.0259 (19)	0.0074 (16)	0.0062 (16)	−0.0039 (15)
Mn7	0.023 (2)	0.0208 (19)	0.0193 (19)	−0.0006 (16)	0.0031 (16)	−0.0021 (15)
Mn8	0.019 (2)	0.0127 (18)	0.015 (2)	−0.0014 (16)	0.0026 (16)	0.0012 (15)

### Geometric parameters (Å)

**Table:**

Mn1—N5	1.632 (14)	C104—C105	1.393 (17)
Mn1—N3	1.937 (12)	C104—H104	0.9500
Mn1—N2	1.950 (12)	C105—C106	1.528 (17)
Mn1—N1	2.026 (12)	C107—C108	1.408 (18)
Mn1—N4	2.069 (12)	C107—C112	1.419 (18)
Mn1—N6	2.132 (12)	C108—C109	1.450 (19)
O1—C6	1.220 (16)	C108—H108	0.9500
O2—C13	1.285 (16)	C109—C110	1.357 (18)
O3—N5	1.211 (15)	C109—H109	0.9500
N1—C1	1.357 (16)	C110—C111	1.393 (18)
N1—C5	1.379 (17)	C110—H110	0.9500
N2—C6	1.357 (17)	C111—C112	1.406 (18)
N2—C7	1.374 (16)	C111—H111	0.9500
N3—C13	1.355 (17)	C113—C114	1.493 (17)
N3—C12	1.402 (16)	C114—C115	1.377 (18)
N4—C18	1.316 (16)	C115—C116	1.407 (18)
N4—C14	1.377 (16)	C115—H115	0.9500
N6—C23	1.345 (17)	C116—C117	1.425 (19)
N6—C19	1.347 (16)	C116—H116	0.9500
N7—C21	1.366 (17)	C117—C118	1.374 (18)
N7—C25	1.436 (17)	C117—H117	0.9500
N7—C24	1.441 (16)	C118—H118	0.9500
C1—C2	1.385 (18)	C119—C120	1.376 (18)
C1—H1	0.9500	C119—H119	0.9500
C2—C3	1.363 (19)	C120—C121	1.414 (17)
C2—H2	0.9500	C120—H120	0.9500
C3—C4	1.384 (17)	C121—C122	1.399 (17)
C3—H3	0.9500	C122—C123	1.360 (18)
C4—C5	1.413 (18)	C122—H122	0.9500
C4—H4	0.9500	C123—H123	0.9500
C5—C6	1.533 (18)	C124—H12D	0.9800
C7—C12	1.421 (18)	C124—H12E	0.9800
C7—C8	1.437 (18)	C124—H12F	0.9800
C8—C9	1.427 (19)	C125—H12G	0.9800
C8—H8	0.9500	C125—H12H	0.9800
C9—C10	1.437 (19)	C125—H12I	0.9800
C9—H9	0.9500	Mn6—N40	1.629 (11)
C10—C11	1.362 (19)	Mn6—N38	1.930 (12)
C10—H10	0.9500	Mn6—N37	1.950 (12)
C11—C12	1.431 (18)	Mn6—N36	2.056 (12)
C11—H11	0.9500	Mn6—N39	2.062 (13)
C13—C14	1.475 (18)	Mn6—N41	2.127 (11)
C14—C15	1.398 (18)	O16—C131	1.229 (16)
C15—C16	1.329 (18)	O17—C138	1.290 (16)
C15—H15	0.9500	O18—N40	1.190 (12)
C16—C17	1.415 (19)	N36—C126	1.345 (18)
C16—H16	0.9500	N36—C130	1.351 (17)
C17—C18	1.360 (18)	N37—C131	1.308 (17)
C17—H17	0.9500	N37—C132	1.390 (16)
C18—H18	0.9500	N38—C138	1.329 (17)
C19—C20	1.349 (18)	N38—C137	1.436 (17)
C19—H19	0.9500	N39—C143	1.331 (17)
C20—C21	1.410 (18)	N39—C139	1.343 (16)
C20—H20	0.9500	N41—C148	1.354 (17)
C21—C22	1.382 (18)	N41—C144	1.355 (16)
C22—C23	1.384 (19)	N42—C146	1.355 (17)
C22—H22	0.9500	N42—C150	1.459 (17)
C23—H23	0.9500	N42—C149	1.475 (17)
C24—H24A	0.9800	C126—C127	1.414 (18)
C24—H24B	0.9800	C126—H126	0.9500
C24—H24C	0.9800	C127—C128	1.41 (2)
C25—H25A	0.9800	C127—H127	0.9500
C25—H25B	0.9800	C128—C129	1.403 (19)
C25—H25C	0.9800	C128—H128	0.9500
Mn2—N12	1.619 (13)	C129—C130	1.437 (18)
Mn2—N9	1.939 (12)	C129—H129	0.9500
Mn2—N10	1.947 (13)	C130—C131	1.526 (18)
Mn2—N11	2.056 (12)	C132—C133	1.366 (17)
Mn2—N8	2.065 (12)	C132—C137	1.432 (17)
Mn2—N13	2.106 (13)	C133—C134	1.417 (18)
O4—C31	1.240 (15)	C133—H133	0.9500
O5—C38	1.266 (16)	C134—C135	1.434 (18)
O6—N12	1.213 (15)	C134—H134	0.9500
N8—C26	1.333 (16)	C135—C136	1.397 (19)
N8—C30	1.366 (16)	C135—H135	0.9500
N9—C31	1.366 (16)	C136—C137	1.424 (18)
N9—C32	1.378 (16)	C136—H136	0.9500
N10—C38	1.301 (16)	C138—C139	1.517 (19)
N10—C37	1.387 (16)	C139—C140	1.389 (18)
N11—C43	1.343 (15)	C140—C141	1.38 (2)
N11—C39	1.375 (16)	C140—H140	0.9500
N13—C44	1.335 (16)	C141—C142	1.417 (19)
N13—C48	1.360 (17)	C141—H141	0.9500
N14—C46	1.364 (16)	C142—C143	1.353 (19)
N14—C50	1.445 (16)	C142—H142	0.9500
N14—C49	1.476 (17)	C143—H143	0.9500
C26—C27	1.390 (18)	C144—C145	1.388 (17)
C26—H26	0.9500	C144—H144	0.9500
C27—C28	1.452 (18)	C145—C146	1.395 (18)
C27—H27	0.9500	C145—H145	0.9500
C28—C29	1.390 (18)	C146—C147	1.383 (19)
C28—H28	0.9500	C147—C148	1.374 (17)
C29—C30	1.408 (18)	C147—H147	0.9500
C29—H29	0.9500	C148—H148	0.9500
C30—C31	1.535 (18)	C149—H14J	0.9800
C32—C33	1.426 (17)	C149—H14K	0.9800
C32—C37	1.429 (18)	C149—H14L	0.9800
C33—C34	1.403 (19)	C150—H15B	0.9800
C33—H33	0.9500	C150—H15C	0.9800
C34—C35	1.351 (18)	C150—H15D	0.9800
C34—H34	0.9500	Mn7—N47	1.679 (12)
C35—C36	1.424 (19)	Mn7—N45	1.952 (13)
C35—H35	0.9500	Mn7—N44	1.997 (13)
C36—C37	1.430 (18)	Mn7—N46	2.076 (12)
C36—H36A	0.9500	Mn7—N43	2.078 (12)
C38—C39	1.456 (17)	Mn7—N48	2.135 (11)
C39—C40	1.376 (18)	O19—C156	1.222 (16)
C40—C41	1.358 (18)	O20—C163	1.259 (16)
C40—H40	0.9500	O21—N47	1.165 (13)
C41—C42	1.399 (19)	N43—C151	1.347 (17)
C41—H41	0.9500	N43—C155	1.383 (16)
C42—C43	1.395 (17)	N44—C156	1.328 (17)
C42—H4A	0.9500	N44—C157	1.417 (17)
C43—H43	0.9500	N45—C163	1.346 (16)
C44—C45	1.383 (18)	N45—C162	1.429 (16)
C44—H44	0.9500	N46—C168	1.361 (17)
C45—C46	1.374 (17)	N46—C164	1.395 (17)
C45—H45	0.9500	N48—C173	1.345 (16)
C46—C47	1.430 (17)	N48—C169	1.375 (17)
C47—C48	1.356 (18)	N49—C171	1.378 (16)
C47—H47	0.9500	N49—C175	1.459 (16)
C48—H48	0.9500	N49—C174	1.464 (16)
C49—H49A	0.9800	C151—C152	1.359 (18)
C49—H49B	0.9800	C151—H151	0.9500
C49—H49C	0.9800	C152—C153	1.391 (19)
C50—H50A	0.9800	C152—H152	0.9500
C50—H50B	0.9800	C153—C154	1.370 (19)
C50—H50C	0.9800	C153—H153	0.9500
Mn3—N19	1.624 (13)	C154—C155	1.394 (18)
Mn3—N16	1.940 (13)	C154—H154	0.9500
Mn3—N17	1.978 (13)	C155—C156	1.537 (19)
Mn3—N18	2.093 (12)	C157—C162	1.418 (17)
Mn3—N15	2.099 (12)	C157—C158	1.434 (18)
Mn3—N20	2.143 (12)	C158—C159	1.425 (19)
O7—C56	1.260 (17)	C158—H158	0.9500
O8—C63	1.294 (17)	C159—C160	1.400 (18)
O9—N19	1.213 (14)	C159—H159	0.9500
N15—C51	1.340 (18)	C160—C161	1.388 (19)
N15—C55	1.387 (18)	C160—H160	0.9500
N16—C56	1.335 (17)	C161—C162	1.420 (18)
N16—C57	1.443 (18)	C161—H161	0.9500
N17—C63	1.306 (17)	C163—C164	1.502 (18)
N17—C62	1.430 (18)	C164—C165	1.414 (18)
N18—C68	1.337 (17)	C165—C166	1.36 (2)
N18—C64	1.387 (17)	C165—H165	0.9500
N20—C69	1.337 (17)	C166—C167	1.358 (19)
N20—C73	1.373 (17)	C166—H166	0.9500
N21—C71	1.368 (15)	C167—C168	1.396 (18)
N21—C74	1.431 (17)	C167—H167	0.9500
N21—C75	1.461 (17)	C168—H168	0.9500
C51—C52	1.410 (18)	C169—C170	1.345 (17)
C51—H51	0.9500	C169—H169	0.9500
C52—C53	1.419 (19)	C170—C171	1.400 (19)
C52—H52	0.9500	C170—H170	0.9500
C53—C54	1.375 (19)	C171—C172	1.383 (18)
C53—H53	0.9500	C172—C173	1.351 (16)
C54—C55	1.430 (19)	C172—H172	0.9500
C54—H54	0.9500	C173—H173	0.9500
C55—C56	1.523 (19)	C174—H174	0.9800
C57—C58	1.41 (2)	C174—H17F	0.9800
C57—C62	1.451 (19)	C174—H17G	0.9800
C58—C59	1.43 (2)	C175—H17H	0.9800
C58—H58	0.9500	C175—H17I	0.9800
C59—C60	1.40 (2)	C175—H17J	0.9800
C59—H59	0.9500	Mn8—N54	1.611 (12)
C60—C61	1.40 (2)	Mn8—N52	1.951 (12)
C60—H60	0.9500	Mn8—N51	1.957 (12)
C61—C62	1.45 (2)	Mn8—N50	2.056 (13)
C61—H61	0.9500	Mn8—N53	2.062 (12)
C63—C64	1.486 (19)	Mn8—N55	2.108 (11)
C64—C65	1.367 (18)	O22—C181	1.249 (16)
C65—C66	1.39 (2)	O23—C188	1.267 (17)
C65—H65	0.9500	O24—N54	1.215 (14)
C66—C67	1.404 (19)	N50—C176	1.330 (17)
C66—H66	0.9500	N50—C180	1.361 (17)
C67—C68	1.370 (18)	N51—C181	1.350 (17)
C67—H67	0.9500	N51—C182	1.428 (16)
C68—H68	0.9500	N52—C188	1.311 (17)
C69—C70	1.357 (17)	N52—C187	1.424 (17)
C69—H69	0.9500	N53—C193	1.318 (17)
C70—C71	1.381 (18)	N53—C189	1.367 (17)
C70—H70	0.9500	N55—C198	1.338 (16)
C71—C72	1.431 (17)	N55—C194	1.366 (16)
C72—C73	1.374 (17)	N56—C196	1.357 (16)
C72—H72	0.9500	N56—C200	1.450 (17)
C73—H73	0.9500	N56—C199	1.474 (17)
C74—H74A	0.9800	C176—C177	1.363 (18)
C74—H74B	0.9800	C176—H176	0.9500
C74—H74C	0.9800	C177—C178	1.442 (18)
C75—H75A	0.9800	C177—H177	0.9500
C75—H75B	0.9800	C178—C179	1.389 (18)
C75—H75C	0.9800	C178—H178	0.9500
Mn4—N26	1.637 (14)	C179—C180	1.423 (17)
Mn4—N24	1.947 (12)	C179—H179	0.9500
Mn4—N23	1.951 (12)	C180—C181	1.537 (19)
Mn4—N22	2.040 (12)	C182—C183	1.419 (17)
Mn4—N25	2.075 (12)	C182—C187	1.425 (17)
Mn4—N27	2.126 (13)	C183—C184	1.392 (19)
O10—C81	1.243 (16)	C183—H183	0.9500
O11—C88	1.263 (17)	C184—C185	1.386 (18)
O12—N26	1.204 (15)	C184—H18D	0.9500
N22—C80	1.336 (17)	C185—C186	1.366 (18)
N22—C76	1.346 (16)	C185—H185	0.9500
N23—C81	1.342 (16)	C186—C187	1.404 (18)
N23—C82	1.398 (16)	C186—H186	0.9500
N24—C88	1.337 (17)	C188—C189	1.501 (18)
N24—C87	1.396 (16)	C189—C190	1.334 (18)
N25—C93	1.305 (17)	C190—C191	1.388 (19)
N25—C89	1.367 (17)	C190—H190	0.9500
N27—C98	1.308 (17)	C191—C192	1.402 (19)
N27—C94	1.358 (16)	C191—H191	0.9500
N28—C96	1.331 (17)	C192—C193	1.382 (18)
N28—C99	1.432 (17)	C192—H192	0.9500
N28—C100	1.473 (17)	C193—H193	0.9500
C76—C77	1.415 (18)	C194—C195	1.363 (17)
C76—H76	0.9500	C194—H194	0.9500
C77—C78	1.386 (18)	C195—C196	1.407 (18)
C77—H77	0.9500	C195—H195	0.9500
C78—C79	1.374 (17)	C196—C197	1.398 (18)
C78—H78	0.9500	C197—C198	1.383 (17)
C79—C80	1.433 (18)	C197—H197	0.9500
C79—H79	0.9500	C198—H198	0.9500
C80—C81	1.491 (18)	C199—H19J	0.9800
C82—C83	1.398 (17)	C199—H19K	0.9800
C82—C87	1.430 (17)	C199—H19L	0.9800
C83—C84	1.404 (18)	C200—H20A	0.9800
C83—H83	0.9500	C200—H20B	0.9800
C84—C85	1.372 (18)	C200—H20C	0.9800
C84—H84	0.9500	O25—C201	1.4297
C85—C86	1.416 (18)	O25—H20D	0.9641
C85—H85	0.9500	C201—C202	1.5153
C86—C87	1.425 (18)	C201—H20E	1.0990
C86—H86	0.9500	C201—H20F	1.0990
C88—C89	1.474 (18)	C202—H20G	1.0939
C89—C90	1.347 (18)	C202—H20H	1.0928
C90—C91	1.395 (18)	C202—H20I	1.0927
C90—H90	0.9500	O26—C203	1.4297
C91—C92	1.411 (18)	O26—H20J	0.8491
C91—H91	0.9500	C203—C204	1.5153
C92—C93	1.387 (18)	C203—H20K	1.0991
C92—H92	0.9500	C203—H20L	1.0990
C93—H93	0.9500	C204—H20M	1.0940
C94—C95	1.369 (18)	C204—H20N	1.0927
C94—H94	0.9500	C204—H20O	1.0928
C95—C96	1.411 (18)	O27—C205	1.4298
C95—H95	0.9500	O27—H20P	0.8400
C96—C97	1.393 (17)	C205—C206	1.5153
C97—C98	1.361 (18)	C205—H20Q	1.0990
C97—H97	0.9500	C205—H20R	1.0990
C98—H98	0.9500	C206—H20S	1.0940
C99—H99A	0.9800	C206—H20T	1.0927
C99—H99B	0.9800	C206—H20U	1.0928
C99—H99C	0.9800	O28—C207	1.4297
C100—H10B	0.9800	O28—H20V	0.8521
C100—H10C	0.9800	C207—C208	1.5153
C100—H10D	0.9800	C207—H20W	1.0991
Mn5—N33	1.624 (14)	C207—H20X	1.0989
Mn5—N30	1.934 (12)	C208—H20Y	1.0940
Mn5—N31	1.950 (12)	C208—H21A	1.0928
Mn5—N32	2.043 (12)	C208—H21B	1.0928
Mn5—N29	2.061 (13)	O29—C209	1.4298
Mn5—N34	2.119 (13)	O29—H21C	0.8400
O13—C106	1.217 (15)	C209—C21F	1.3350
O14—C113	1.277 (16)	C209—H21D	0.9900
O15—N33	1.205 (15)	C209—H21E	0.9900
N29—C101	1.298 (16)	C21F—H21G	0.9800
N29—C105	1.363 (17)	C21F—H21H	0.9800
N30—C106	1.334 (16)	C21F—H21I	0.9800
N30—C107	1.438 (16)	O30—C211	1.4297
N31—C113	1.350 (17)	O30—H21J	0.8505
N31—C112	1.379 (16)	C211—C212	1.5152
N32—C118	1.316 (16)	C211—H21K	1.0990
N32—C114	1.369 (17)	C211—H21L	1.0990
N34—C119	1.327 (17)	C212—H21M	1.0940
N34—C123	1.370 (16)	C212—H21N	1.0928
N35—C121	1.354 (16)	C212—H21O	1.0928
N35—C125	1.443 (16)	O31—C213	1.4297
N35—C124	1.456 (16)	O31—H21P	0.8497
C101—C102	1.419 (18)	C213—C214	1.5153
C101—H101	0.9500	C213—H21Q	1.0990
C102—C103	1.397 (17)	C213—H21R	1.0991
C102—H102	0.9500	C214—H21S	1.0939
C103—C104	1.388 (17)	C214—H21T	1.0928
C103—H103	0.9500	C214—H21U	1.0928

### Hydrogen-bond geometry (Å, °)

**Table:**

*D*—H···*A*	*D*—H	H···*A*	*D*···*A*	*D*—H···*A*
O25—H20D···O22^i^	0.96	1.88	2.751 (15)	149.
O26—H20J···O8^ii^	0.85	1.84	2.686 (17)	178.
O27—H20P···O16^iii^	0.84	1.93	2.739 (18)	161.
O28—H20V···O23	0.85	1.89	2.747 (19)	179.
O29—H21C···O20	0.84	1.93	2.709 (17)	154.
O30—H21J···O7^iv^	0.85	1.90	2.75 (2)	178.
O31—H21P···O13	0.85	2.04	2.887 (17)	179.

Symmetry codes: (i) *x*, *y*−1, *z*; (ii) *x*, −*y*, *z*+1/2; (iii) *x*, *y*+1, *z*; (iv) *x*, −*y*+1, *z*+1/2.
